# Association of sleep duration and sleep quality with cognitive frailty in Chinese older adults

**DOI:** 10.3389/fpubh.2025.1596965

**Published:** 2025-06-06

**Authors:** Jinqiang Qian, Xin Yu, Qiang Zhang

**Affiliations:** ^1^Department of Geriatrics, Tianjin Medical University General Hospital, Tianjin Key Laboratory of Elderly Health, Tianjin Geriatrics Institute, Tianjin, China; ^2^Department of Nephrology, Tianjin People's Hospital, Tianjin, China

**Keywords:** sleep duration, sleep quality, napping time, risk factor, cognitive frailty

## Abstract

**Objective:**

To explore the relationship between sleep duration, sleep quality and the occurrence of cognitive frailty in the older adults.

**Methods:**

A total of 9,970 participates were screened in China over the past 9 years. They were divided into cognitive frailty group and non-cognitive frailty groups, and they were evaluated for sleep duration and sleep quality and their relationship with cognitive frailty was analyzed. If interactions are found, further hierarchical analysis is conducted.

**Results:**

One thousand six hundred eighty-four participants (16.89%) were diagnosed with cognitive frailty. Participants with cognitive frailty were more likely to be “unmarried,” live in rural areas, and were female, with no social activity in the last month. Poor sleep quality, short sleep duration, no napping, and excessive napping are at high risk of cognitive frailty. There was a significant interaction between daytime napping and sleep duration and sleep quality. Among participants with good sleep quality, those who took excessive naps had a 123% increased risk of developing cognitive frailty, with an OR of 2.23 (95% CI: 1.72, 2.86). In the subgroup with sleep duration > 9 h, participants who napped excessively had a significantly increased risk of cognitive frailty (OR = 1.62, 95% CI 1.14–2.30, *p* < 0.001).

**Conclusion:**

Chinese older adults with poor sleep quality are at a 67% higher risk of cognitive weakness, and individuals with less than 6 h of sleep are at a 48% higher risk of cognitive weakness; No napping and excessive napping, the risk of cognitive debilitation increased by 23 and 69%, respectively. There is an additive interaction between sleep duration and quality and daytime napping on cognitive frailty in the older adults.

## 1 Introduction

Population aging has become a common phenomenon worldwide, and the average life expectancy of Chinese is also increasing year by year (1, 2). Cognitive impairment and frailty have become major threats to healthy ageing and the quality of life of the older adults (3, 4), and there is an interaction between them, which accelerates the decline of physical functions (5). Therefore, the International Academy on Nutrition and Aging (IANA) and the International Association of Gerontology and Geriatrics (IAGG) formally proposed the concept of “cognitive frailty” in 2013. refers to the presence of both physical frailty and cognitive impairment and excludes dementia and other neurodegenerative diseases (6).

Cognitive frailty has been reported to increase the incidence of dementia (7) and is associated with adverse health outcomes such as decreased functioning, disability, poor quality of life (8), and increased mortality (9). It is generally accepted that the prevalence is higher in older age, females, and people with low educational attainment (10–14). Some scholars have also explored the influencing factors in terms of lifestyle behavior, dietary nutrition, and mental health status, but the conclusions of the study mainly rely on cross-sectional surveys12,15 (13, 15).

Sleep constitutes a significant portion of our daily routine, however, there has been considerable changes in the average sleep duration of population in recent years (16). About one-third of the adults in the United States reported obtaining less than 7 h of daily sleep (17).

Both short and long sleep durations are associated to increased risk of major health problems, including diabetes, cardiovascular disease, and mortality (18, 19). And there is growing evidence that sleep affects the risk of cardiovascular disease (20).

As older adults age, they may experience decreased nighttime sleep (21), poorer sleep efficiency and continuity (22), more frequent nocturnal awakenings (23), less time spent in slow-wave sleep and REM sleep (24), more fragmented sleep, and a faster sleep-to-wake transition (25), and these changes may also lead to changes in brain function (26). Sleep disturbance leads to the development of cognitive dysfunction (27) and accelerates the loss of nerve cells in the frontal, parietal, and temporal lobes of the brain, affecting synaptic plasticity and neuronal function, leading to cognitive impairment or dementia (28, 29). Studies have shown that changes in sleep parameters are associated with cognitive decline (30), and cross-sectional studies have found that the rate of cognitive decline in patients with sleep disorders is 2–4 times higher than that of those without sleep disorders (31). Therefore, sleep disturbance is seen as a possible potential trigger or biomarker of cognitive alteration, and monitoring sleep quality is promising as a non-invasive means to assess the risk of future Alzheimer’s disease (AD) or to monitor the effects of clinical interventions (32).

The concept of sleep deprivation is often involved in studying the link with cognition. In many studies, shortened sleep is considered total sleep deprivation (TSD), a useful indicator to comprehensively investigate how shortened sleep harms cognition (33). As the number of sleep-deprived people continues to grow, it is important to understand how sleep deprivation affects human cognitive function to prevent its negative effects. In particular, people in many fields of work are affected by reduced sleep duration, which puts them at higher risk while working. For example, shortened sleep duration is a significant cause of industrial and transportation-related accidents (34). Therefore, exploring how sleep deprivation affects human cognitive function is essential to prevent its harmful effects. Previous studies have shown that TSD impairs different levels of cognitive function. For example, TSD significantly impairs attentional performance (35–38), and TSD also impairs higher cognitive processes and even social cognitive abilities (39, 40).

To sum up, we can see that sleep and cognitive frailty are closely related. Previous studies have mostly independently studied the relationship between sleep and cognitive function or frailty (41–43), but there is still a gap in the research on sleep and cognitive frailty, and the relationship between sleep duration, sleep quality and cognitive frailty in the older adults is not very clear, which also provides space for this study to explore. This study aims to explore the relationship between sleep duration, sleep quality and cognitive frailty in the older adults.

## 2 Materials and methods

### 2.1 Study design and participants

**Figure 1:**
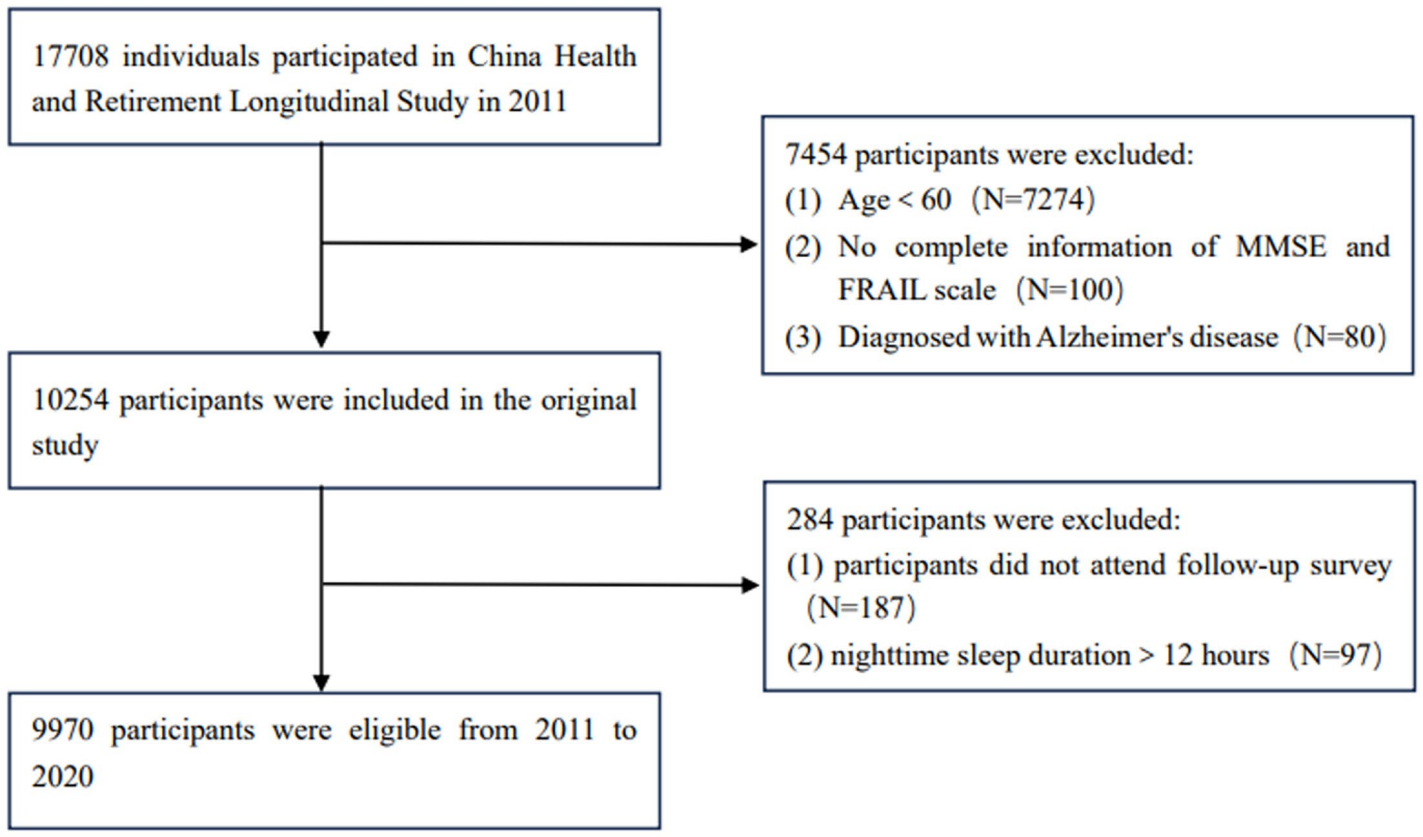
The flow chart of study population.

The data for this study were obtained from the China Health and Elderly Care Longitudinal Survey (CHARLS), which was approved by the Biomedical Ethics Committee of Peking University (IRB00001052-11015) in 2011. The project is a nationally representative cohort study that comprehensively investigates information from multiple perspectives, including basic information, health status and function information, personal income, household income and expenditure. The project was evaluated every 2 years, and the latest round of data was released on 2023-11-16. This study was a nine-year longitudinal study based on 2011 data, with 17,708 participants initially recruited and 9,970 participants included in the analysis. The enrollment criteria were: (1) seniors aged 60 years and above; (2) The data items required by the research subjects are complete and accurate. The exclusion criteria were: (1) No complete information of cognitive function questionnaire and FRAIL scale; (2) Those who did not have successful follow-up. The inclusion flowchart is shown in Figure 1.

### 2.2 Measurements

#### 2.2.1 Assessment of napping time

Participants were asked to describe their nap habits by answering the following question: “Are you in the habit of taking daytime naps? Those who reported “no” were classified as non-nappers, while others who reported “yes” were classified as nappers. For nappers, napping duration was categorized as ≤ 60 min/day, 61–90 min/day, and > 90 min/day.

#### 2.2.2 Assessment of sleep quality and duration

The sleep duration was derived from self-reports by participants, collected by asking the following question regarding nocturnal sleep duration: “In the previous month, how many hours of actual sleep did you get each night?” Based on previous research (44, 45), night-time sleep duration was divided into short (≤ 6 h), medium (6–9 h), and long (> 9 h).

Sleep quality was also self-reported by participants, determined by their response to the question, “How many days of restless sleep did you have in the last week?” The response options encompassed four categories: (1) rarely or none of the time (<1 d/week); (2) some or a little of the time (1–2 days/week); (3) occasionally or a moderate amount of the time (3–4 days/week); and (4) most or all of the time (5–7 days/week). We classified (1) as good sleep quality, (2) and (3) as moderate sleep quality, and (4) as poor sleep quality drawing upon prior studies (46).

The above sleep questionnaire is shown in Supplementary Table 1.

#### 2.2.3 Assessment of cognitive frailty

##### 2.2.3.1 Physic frail

Physical frailty was assessed using the modified version of the Fried’s Frailty Phenotype (47), which has been widely used among older PWH populations. The tool includes 5 components with self-report and objective measurements: unintentional weight loss, self-reported low physical activity, self-reported exhaustion, weak grip strength, and slow gait speed. Measurement details for each component have been published and described elsewhere. Participants who met0 components were classified as robust, 1 or 2 components as refrailty, and 3 or more components as frailty.

##### 2.2.3.2 Cognitive function

CHARLS’s assessment of cognitive function includes self-reported assessment of cognitive decline, telephone interview on cognitive status (TICS-10), word recall, and graphic drawing. The TICS scale measures an individual’s cognitive function in two dimensions: memory and mental state (48). Memory is measured by testing the respondent’s ability to recall words immediately (0–10 points) and delayed word recall (0–10 points), and respondents are asked to recall and repeat them, and respondents are able to repeat a word for a short period of time or after an interval of 1 point. Mental state is measured from three aspects, including orientation, visual construction, and mathematical performance. The cognitive function score is calculated by summing the scores of the above questions, with a total score ranging from 0 to 31, with higher scores indicating better cognitive function and more complete cognitive ability (49). The cutoff value was set at 18 for illiterate individuals, 21 for individuals with 1–6 years of education, and 25 for individuals with seven or more years of education, and cognitive impairment was defined as those whose score was lower than the cutoff value according to their education level.

##### 2.2.3.3 Cognitive frailty

In line with the definition by (I. A. N. A/I. A. G. G) international consensus group. I.e. as mentioned above, cognitive frailty was defined as the simultaneous present of both cognitive impairment and physical frailty, which has been validated in previous studies (50, 51).

#### 2.2.4 Assessment of covariates

According to the previous literature (49, 52–57), potential confounding factors were included as covariates in this study, mainly including age, sex (male and female), level of education (elementary school, junior high school, high school, university), marital status (married and unmarried), and place of residence (rural and urban), smoking and drinking, all of which can be obtained from the Demographic Background section of CHARLS. Body mass index (BMI) is calculated by dividing your weight by the square of your height. Chronic disease refers to a person with any of the 14 diseases in the DA007 answers in the CHARLS Household Questionnaire.

### 2.3 Statistical analysis

Continuous variables were expressed as mean ± standard deviation, while categorical variables were described using rates and proportions. The Kruskal–Wallis H test was applied to continuous variables, and chi-squared tests were used for categorical variables. We employed multivariate logistic regression analysis to explore the relationship between sleep and cognitive frailty. Three models were constructed for this purpose by adjusting for potential confounding factors. The results are presented as odds ratios (OR) with 95% confidence intervals (95% CI). Subgroup analyses stratified by interaction analyses were performed. Restricted cubic spline (RCS) was utilized to further explore the relationship between sleep duration and cognitive frailty risk. The significance level of the statistical tests was set as *p* < 0.05.

## 3 Results

### 3.1 Characteristics of 9,970 participants grouped by whether they were diagnosed with cognitive frailty

In this study, 1,684 participants were defined as cognitive frailty, accounting for 16.89%, with a median age of 68 years, 48.56% of participants were male and 51.44% were female. Participants with cognitive frailty were more likely to be “unmarried,” live in rural areas, and were female, with no social activity in the last month (see Table 1).

**Table 1:** Characteristics of 9,970 participants by CF group and non-CF group.

Item	Total	CF	Non-CF	*P*
N	9,970	1,684	8,286	-
Age (years), mean (SD)	67 (63,71)	68 (64,72)	66 (63,70)	<0.0001
Gender, *n*%				<0.0001
Male	4,841 (48.6)	690 (40.1)	4,151 (50.1)	
Female	5,129 (51.4)	994 (59.9)	4,135 (49.9)	
Education level				<0.0001
Elementary school	2,884 (28.9)	806 (47.9)	2078 (25.1)	
Junior high school	4,313 (43.3)	434 (25.8)	3,879 (46.8)	
High school	1,362 (13.7)	276 (16.4)	1,086 (13.1)	
University	1,411 (14.1)	168 (9.9)	1,243 (15)	
Area of residence				<0.0001
Urban	4,279 (42.9)	624 (37.1)	3,655 (44.1)	
Rural	5,691 (57.1)	1,060 (62.9)	4,631 (55.9)	
Current marital status, *n*%				<0.0001
Not married	1,618 (16.2)	325 (19.3)	1,293 (15.6)	
Married or cohabitated	8,352 (83.8)	1,359 (80.7)	6,993 (84.4)	
BMI(kg/m^2^)				<0.0001
< 18.5	4,156 (41.7)	818 (48.6)	3,338 (40.3)	
18.5–23.9	4,380 (44.0)	595 (35.3)	3,785 (45.7)	
≥ 24	1,434 (14.3)	271 (16.1)	1,163 (15.0)	
Drinking				0.053
Never	4,656 (46.7)	767 (45.5)	3,889 (46.9)	
Former	683 (6.9)	234 (13.9)	449 (7.9)	
Current	4,431 (44.4)	683 (40.6)	3,748 (45.2)	
Smoking				0.041
Never	7,886 (79.1)	1,315 (78.1)	6,571 (79.3)	
Former	1,172 (11.8)	211 (12.5)	961 (11.6)	
Current	912 (9.1)	158 (9.4)	754 (9.1)	
Family income (anu/year), %				<0.0001
< 10,000	1,254 (12.5)	310 (18.4)	944 (11.4)	
10,000–19,999	1881 (18.9)	497 (29.5)	1,384 (16.7)	
≥ 20,000	6,835 (68.6)	877 (52.1)	5,958 (71.9)	
Social interaction				0.0005
Never	6,196 (62.1)	918 (54.5)	5,278 (63.7)	
At least once a month	3,774 (37.9)	766 (45.5)	3,008 (36.3)	
Numbers of chronic diseases				0.324
≤1	2,919 (29.3)	317 (18.8)	2,602 (31.4)	
2–3	2,235 (22.4)	495 (29.4)	1740 (21.0)	
≥4	4,816 (48.3)	872 (51.8)	3,944 (47.6)	
Depression	1,611 (16.2)	527 (31.3)	655 (7.9)	<0.0001
Daytime napping	30 (0,60)	40 (0,60)	30 (0,60)	<0.0001
Sleep duration	6 (5,7)	6 (5,8)	6 (5,7)	0.042
Sleep quality				<0.0001
Good	2,433 (24.4)	295 (17.5)	2,138 (25.8)	
Moderate	2,382 (23.9)	443 (26.3)	1939 (23.4)	
Poor	5,155 (51.7)	946 (56.2)	4,209 (50.8)	

CF: cognitive frailty.

### 3.2 Prevalence and adjusted OR (95% CI) of cognitive frailty by groups of napping time, sleep duration and quality

Table 2 presents that individuals who sleep less than 6 h have a significantly higher risk of cognitive frailty. This finding was confirmed in different models. Specifically, in Model 1, those who slept less than 6 h had a 55% increased risk of developing cognitive frailty compared to those who slept 6 to 9 h (OR = 1.55, 95% CI: 1.36, 1.77); In fully adjusted model 2, those who slept less than 6 h had a 48% increased risk of developing cognitive frailty (OR = 1.48, 95% CI: 1.25, 1.64); In Model 3, which was further adjusted for sleep quality or daytime napping, those who slept less than 6 h had a 27% increased risk of developing cognitive frailty (OR = 1.27, 95% CI: 1.13, 1.49); Conversely, no significant differences were observed in participants who slept more than 9 h across the models (model 1: OR = 1.03, 95% CI: 0.88, 1.29; model 2: OR = 0.98,95% CI: 0.76,1.19; model 3: OR = 1.27,95% CI: 0.68,1.17).

**Table 2:** Prevalence and adjusted OR (95% CI) of cognitive frailty by groups of napping time, sleep duration and quality.

Variables	Model 1	Model 2	Model 3
Sleep quality			
Good	Ref	Ref	Ref
moderate	1.36 (1.18,1.56)	1.57 (1.38,1.79)	1.68 (1.48,1.89)
Poor	1.38 (1.14,1.57) ***	1.67 (1.47,1.95) ***	1.88 (1.58,2.19) ***
Napping time			
60-90 min	Ref	Ref	Ref
Non-nappers	1.28 (1.13,1.46)	1.25 (1.04,1.47)	1.23 (1.04,1.46) ***
< 60 min	1.23 (1.08,1.59)	1.21 (1.06,1.56)	1.19 (1.02,1.48)
> 90 min	1.71 (1.35,2.12)	1.68 (1.31,2.17)	1.69 (1.38,2.19) ***
Sleep duration			
6–9 h	Ref	Ref	Ref
< 6 h	1.55 (1.36,1.77) ***	1.48 (1.25,1.64) ***	1.27 (1.13,1.49) ***
> 9 h	1.03 (0.88,1.29)	0.98 (0.76,1.19)	0.96 (0.68,1.17)

Model 1: adjusting for sex, age, education level, residence, current marital status, personal income, smoking status, alcohol consumption, social interaction; Model 2: additionally adjusting for BMI, numbers of chronic diseases; Model 3: additionally adjusting for daytime napping or nighttime sleep quality. *p < 0.05, **p < 0.01, ***p < 0.001.

Regarding sleep quality, in Model 1, individuals with poor sleep quality had a 38% increased risk of developing cognitive frailty compared to those with good sleep quality (OR = 1.38, 95%CI: 1.14, 1.57). This trend was further confirmed by adjusting for all covariates in Model 2. In addition, after adjusting for potential confounders and napping (model 3), people with poor sleep quality had a higher chance of developing cognitive frailty [OR 1.88 (95% CI: 1.58, 2.19)].

About the nap aspect, after further adjustment for sleep quality (Model 3), participants who did not nap and who took excessive naps for > 90 min/day had high rates of cognitive frailty, with an OR (95% CI) of 1.23 (1.04 to 1.46) and 1.69 (1.38 to 2.19), respectively.

### 3.3 Additive interactive effect of sleep quality, sleep duration and napping time on cognitive frailty in older adults people based on multivariate logistic regression analysis

Table 3 shows that Multivariate Logistic regression analysis was conducted with the older adults cognitive frailty as the dependent variable, sleep quality, Sleep duration and napping time as independent variables, and controlling for confounding factors such as age, education level, residence, and the number of chronic diseases. The results showed that there was an interaction among sleep quality, sleep duration and siesta on cognitive frailty in the older adults, and the *p* values of the interaction were 0.035, 0.042 and 0.005, respectively. Specifically, the coefficient for the interaction between sleep duration and sleep quality was 0.728 (SE = 0.793, *p* = 0.035), suggesting a significant interaction between sleep duration and sleep quality on depression (95% CI: 0.631, 2.585). The coefficient of the interaction between sleep quality and napping was 0.896 (SE = 1.485, *p* = 0.042), suggesting that there was a significant interaction between sleep quality and napping on cognitive frailty (95% CI: 0.802, 2.248). The coefficient of the interaction between sleep duration and napping is 0.963 (SE = 1.129, *p* = 0.005), suggesting a significant interaction between sleep quality and napping on cognitive frailty (95%CI: 1.022, 2.748). Co-linearity diagnosis: VIF of all variables is less than3, indicating that the multicollinearity problem is negligible. This suggests that these factors do not act independently, but act together in the effects of sleep quality, sleep duration, and napping on cognitive weakness, and it is necessary to further stratify sleep quality, sleep duration, and napping.

**Table 3:** Additive interactive effect of sleep quality, sleep duration and napping time on cognitive frailty in older adults people based on multivariate logistic regression analysis.

Variables	B	SE	Wald χ2	p value	OR(95%CI)	VIF
Sleep quality X Sleep duration	0.728	0.793	0.048	0.035	1.535 (0.631, 2.585)	2.0
Sleep quality X Napping time	0.896	1.485	0.082	0.042	1.347 (0.802, 2.248)	2.3
Sleep duration X Napping time	0.963	1.129	0.384	0.005	1.476 (1.022, 2.874)	1.6

### 3.4 Joint effect of sleep duration and sleep quality on cognitive frailty

Table 4 displays that due to the interaction between sleep duration and sleep quality on cognitive weakness in the older adults (*β* = 0.728, 95% CI: 0.631 ~ 2.583, *p* = 0.035). When the sleep quality is poor, the protective effect of prolonged sleep time on cognition is weakened. Model multicollinearity acceptable (VIF = 2.0). We observe that individuals with short sleep duration have a higher risk of developing CF, regardless of whether they sleep well or poorly. However, people with short sleep duration and poor sleep quality have a 65% increased risk of cognitive frailty. The individuals who slept for long periods of time did not show a similar increased risk.

**Table 4:** Joint effect of sleep duration and sleep quality on cognitive frailty (*n* = 1,684).

Variables	Model 1	Model 2	Model 3
Duration quality			
6–9 h good	Ref	Ref	Ref
<6 h good	2.09 (1.65, 2.32) ***	1.85 (1.36, 2.01) ***	1.45 (1.14, 1.85) ***
<6 h poor	2.12 (1.74, 2.48) ***	1.90 (1.48, 2.23) ***	1.65 (1.34, 2.15) ***
>9 h good	1.62 (1.23, 2.34)	1.52 (0.98, 2.33)	1.25 (0.74, 1.95)
>9 h poor	1.42 (0.84, 2.88)	1.20 (0.58, 2.63)	1.05 (0.64, 2.35)

Model 1: adjusting for sex, age, education level, residence, current marital status, personal income, smoking stat-us, alcohol consumption, social interaction; Model 2: additionally adjusting for BMI, numbers of chronic diseases; Model 3: additionally adjusting for daytime napping or nighttime sleep quality. *P < 0.05, **P < 0.01, ***P < 0.001.

### 3.5 Joint effect of daytime napping duration and sleep quality on cognitive frailty

Since we found a significant interaction between daytime napping and sleep quality (*β* = 0.896, 95% CI: 0.802 ~ 2.248, *p* = 0.042), a stratified analysis by sleep quality (Table 5) showed a 123% increased risk of developing cognitive frailty in participants with good sleep quality who napped for more than 90 min, with an OR of 2.23 (95% CI: 1.72 ~ 2.86). No association was found between napping and cognitive frailty in participants with poor sleep quality. The model multicollinearity is acceptable (VIF = 2.3).

**Table 5:** Joint effect of daytime napping duration and sleep quality on cognitive frailty (*n* = 1,684).

Variables	Model 1	Model 2
Good sleep quality		
None	Ref	Ref
< 60 min	1.86 (1.36,2.25)	1.68 (1.22,2.06)
60-90 min	2.04 (1.62,2.96)**	1.89 (1.03,2.64)**
> 90 min	2.23 (1.72,2.86)***	2.10 (1.63,2.75)***
Poor sleep quality		
None	Ref	Ref
< 60 min	1.16 (0.85,1.48)	1.13 (0.76,1.43)
60-90 min	1.18 (0.79,1.65)	1.36 (0.96,1.79)
> 90 min	1.06 (0.66,1.25)	1.12 (0.71, 1.44)

Model 1: adjusting for sex, age, education level, residence, personal income, smoking status, alcohol consumption, social interaction; Model 2: additionally adjusting for BMI, numbers of chronic diseases. *P* < 0.05, **P < 0.01, ***P < 0.001.

### 3.6 Joint effect of daytime napping duration and nighttime sleep duration on cognitive frailty

The relationship between sleep duration, napping duration and cognitive frailty is shown in Table 6 (*β* = 0.963, 95% CI: 1.022 ~ 2.874, *p* = 0.005). No significant association was found between daytime naps and cognitive impairment in the shorter and moderate nocturnal sleep groups. In the subgroup with longer sleep duration, participants who took excessive naps had a significantly increased risk of cognitive frailty (OR = 1.62, 95% CI: 1.14 ~ 2.30, *p* < 0.001), i.e., excessive napping (> 90 min) was associated with cognitive frailty in participants who slept 9 h or more at night. The model multicollinearity is acceptable (VIF = 1.6).

**Table 6:** Joint effect of daytime napping duration and nighttime sleep duration on cognitive frailty (*n* = 1,684).

Variables	Model 1	Model 2	Model 3
<6 h/nighttime			
None	Ref	Ref	Ref
< 60 min	0.98 (0.48, 1.95)	0.94 (0.40, 1.81)	0.95 (0.53, 2.07)
60-90 min	0.88 (0.37, 1.89)	0.75 (0.42, 1.76)	0.64 (0.45, 1.57)
>90 min	1.14 (0.38, 1.98)	1.17 (0.78, 2.06)	1.06 (0.79, 1.96)
6-9 h/nighttime			
None	Ref	Ref	Ref
< 60 min	0.98 (0.47, 1.83)	0.95 (0.34, 1.93)	0.99 (0.78, 2.17)
61-90 min	0.73 (0.38, 1.66)	0.85 (0.52, 1.86)	0.65 (0.56, 1.85)
>90 min	1.28 (0.59, 2.08)	1.25 (0.69, 1.91)	1.18 (0.91, 2.21)
>9 h/nighttime			
None	Ref	Ref	Ref
<60 min	0.78 (0.33, 1.78)	0.85 (0.36, 1.79)	0.84 (0.65, 1.94)
61-90 min	1.34 (0.56, 1.95)**	1.43 (0.86, 2.21)**	1.56 (1.21, 2.54)**
>90 min	1.46 (0.88, 1.98)***	1.55 (0.91, 2.36)***	1.62 (1.44, 2.60)***

Model 1: adjusting for sex, age, education level, residence, personal income, smoking status, alcohol consumption, social interaction; Model 2: additionally adjusting for BMI, numbers of chronic diseases; Model 3: addition-ally adjusting for daytime napping or nighttime sleep quality. *P* < 0.05, **P < 0.01, ***P < 0.001.

**Figure 2:**
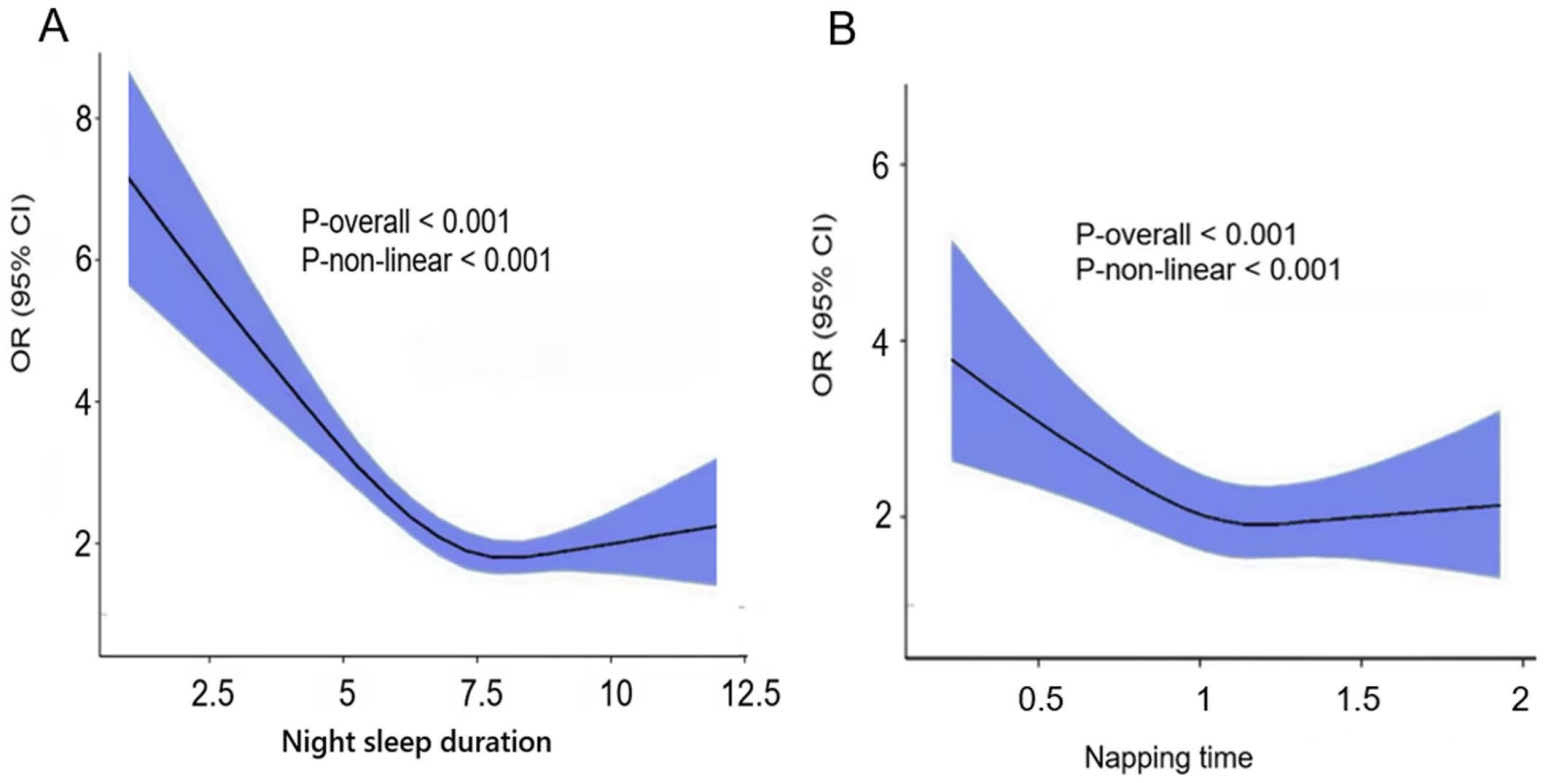
**(A)** Night sleep duration. **(B)** Napping time. Restricted cubic spline of the association between night sleep duration/Napping time and the risk of cognitive frailty. The model was adjusted for age, sex, education level, marital status, residence, alcohol consumption, social interaction, BMI, numbers of chronic diseases.

The study revealed a linear relationship between sleep duration and cognitive frailty, but also detected a nonlinear relationship (P-nonlinearity <0.001). Using a fully adjusted model, restrictive cubic spline regression showed a nonlinear (U-shaped) relationship between nighttime sleep duration and napping time and the risk of cognitive frailty (Figure 2). As sleep duration increased to 7.5 h per night or nap 1.2 h, the risk of cognitive impairment decreased, followed by a reversal trend above these thresholds. Therefore, our study suggests that both too short and too long sleep may increase the risk of cognitive weakness. The results for longer sleep durations in the restricted cubic spline regression differed from the non-significant results for the fully adjusted linear model. When comparing the linear model and the nonlinear model, it is shown that the linear model performs better than the nonlinear model. Therefore, our study tends to conclude that prolonged sleep is unlikely to increase the risk of cognitive weakness. This difference may be attributed to the relatively small sample size in the long sleep duration group, which complicates the interpretation of the results.

### 3.7 Mediation analysis of the association of sleep disorders with cognitive frailty by depression

Based on the significant association between sleep disorder and cognitive frailty observed among older adults in the abovementioned results, a mediation analysis was further conducted to examine whether depression mediated the association between sleep disorder and cognitive frailty (Table 7). The results showed that the association between sleep and risk of cognitive frailty was significantly mediated by depression. After adjusting for potential confounding factors, depression completely mediated the sleep disorder–cognitive frailty association, with a significant indirect effect (OR = 0.995, 95%CI: 0.993 ~ 0.997, *p* < 0.001) but a non-significant direct effect (OR = 1.004, 95% CI: 0.998 ~ 1.012, *p* = 0.278). It can be seen from the mediation analysis revealed that the relationships of poor sleep.

**Table 7:** Mediation analysis of the association of sleep disorders with cognitive frailty by depression.

Variables	Coefficient (95% BCa CI)	OR (95% CI)	Z	*p* value
Sleep quality				
Indirect effect-path ab	0.030 (0.015–0.049)	1.022 (1.009–1.035)	3.453	<0.001
Direct effect–path c’	0.021(−0.006–0.04)	1.034 (1.006–1.060)	2.341	0.159
Direct effect–path c	0.052 (0.020–0.085)	1.059 (1.028–1.093)	3.384	<0.001
Napping time				
Indirect effect–path ab	0.022 (0.011–0.034)	1.030 (1.015–1.048)	3.867	<0.001
Direct effect–path c’	0.023 (0.009–0.071)	1.025 (0.093–1.057)	1.325	0.146
Direct effect–path c	0.053 (1.08,1.59)	1.051 (1.026–1.084)	3.342	<0.001
Sleep duration				
Indirect effect-path ab	−0.004 (−0.006 to −0.002)	0.995 (0.993–0.997)	−4.187	<0.001
Direct effect–path c’	0.005 (−0.001 to 0.011)	1.004 (0.998–1.012)	1.207	0.278
Direct effect–path c	0.001 (−0.005 to 0.009)	1.001 (0.995–1.006)	0.363	0.734

Adjusted for age, gender, smoking, alcohol consumption, Social interaction and chronic diseases. Path ab coefficient represents 5,000 bootstrapped samples and BCa 95% CIs. Path c’: the direct effect of the exposure variable (sleep) on the outcome (cognitive frailty), holding the effect of depression constant. Path c: the total effect of exposure (sleep) on the outcome (cognitive frailty). BCa, bias-corrected and accelerated; OR, odds ratio.

quality and sleep duration with cognitive frailty were mediated by depression in older adults.

## 4 Discussion

This study indicated the prevalence of cognitive frailty was 16.89%. Participants with cognitive frailty were more likely to be “unmarried,” live in rural areas, have low levels of education, have had no social activity in the last month, and have depression. Previously, a study of 7,338 older adults people in the community by Aliberti et al. (58). reported a 5% prevalence of cognitive frailty. In a study of 1,620 older patients over 65 years of age at the Toulouse Frailty Day Hospital, the prevalence of cognitive frailty was as high as 26.7 percent (59). The difference in the detection rate of cognitive frailty may be due to the different age ranges and urban–rural distribution of the survey subjects. In addition, it may also be related to the assessment methods and evaluation tools.

We assessed the association between sleep duration, sleep quality, nap duration, and cognitive frailty after controlling for confounding factors. Participants who slept less than 6 h, no and excessive napping, and poor sleep quality had significantly increased risk of cognitive vulnerability. Previous investigators, Kong et al. (60). using a cross-sectional survey of community-dwelling older adults with type 2 diabetes, found that those who had insufficient sleep at night were at higher risk for cognitive frailty. In contrast, the Leng report showed that men who took excessive naps per day were 66% more likely to have cognitive impairment than those who took 30-min nap (61). In Li′s study, moderate nappers had better overall cognitive performance than those who did not nap or who took long naps (62), which is consistent with the results of this study.

Exploring the mechanisms by which sleep deprivation affects cognitive function is essential to reduce its harm. Complete sleep deprivation (TSD), as an extreme manifestation of sleep deprivation, is a valid model for comprehensively studying how sleep deprivation impairs cognition (63). Numerous studies have shown that TSD impairs executive function. Honn et al. (64) revealed that TSD impairs feedback blunting in cognitive flexibility tasks. Aidman et al. (65) conducted a study showing that TSD impairs three executive functions, namely cognitive flexibility, inhibitory control, and working memory capacity. In addition, Cain et al. (66) found that TSD affected the performance of the Stroop task through an increase in overall response time. In addition, some studies have shown that female cognitive function is less susceptible to the effects of TSD (67, 68), which may be explained by gonadal hormone status (69). Taken together, these studies suggest that cognitive function is negatively affected by TSD. There was a significant interaction among sleep quality, sleep duration and nap duration on cognitive frailty. Specifically, After controlling for confounding factors such as gender, age, education level, and nighttime sleep duration, participants with good sleep quality had a significantly increased risk of excessive napping for cognitive frailty. No association was found between napping and cognitive frailty among participants with poor sleep quality, highlighting the importance of modifiable behavioral factors in preventing cognitive frailty. Individuals with short sleep duration are at higher risk for cognitive weakness, regardless of whether they have good or poor sleep quality. However, individuals with longer sleep duration did not show a similar increased risk. Excessive naps (> 90 min) were associated with increased risk for cognitive frailty in the longer sleep duration subgroup, whereas no significant association was found between daytime naps and cognitive frailty in the shorter and moderate sleep groups. Many researchers have reported that excessive napping during the day may lead to cognitive decline in older adults with or without dementia. Excessive daytime napping has been suggested to increase the risk of Alzheimer’s dementia and is associated with deterioration of cognitive function (70–72).

The underlying mechanisms underlying the association between sleep disturbance and cognitive weakness in patients are poorly understood. The mediation analysis used in this study may provide epidemiological evidence that mediating effects through depression may be an underlying mechanism that influences sleep in relation to cognitive vulnerability. Sleep disturbances (e.g., insomnia and poor sleep quality) are often combined with cognitive weakness in old age. Previous literature has shown that sleep disturbances may lead to depression and reduce physical inactivity (73), thereby increasing the risk of negative health outcomes.

In conclusion, this study systematically reveals for the first time the synergistic effect between sleep quality, nighttime sleep duration, and napping behavior on cognitive frailty in the older adults population. Different from previous studies that only focused on a single sleep factor, we broke through the limitations of traditional logistic regression by introducing interaction terms and hierarchical analysis, and constructed a multi-dimensional dynamic model including sleep quality, duration, and napping. Our stratified analysis revealed a significant association between sleep disorders (including long naps, poor sleep quality, short sleep duration, and long sleep duration) and cognitive frailty in Chinese older adults. For example, the interaction effect analysis of ‘sleep quality ×nap duration’ (*p* = 0.042) revealed the moderating effect of the temporal distribution of sleep behavior on cognitive frailty. Participants with good sleep quality who took naps for more than 90 min had a 123% increased risk of cognitive frailty and an OR of 2.23 (95% CI: 1.72 ~ 2.86). These results suggest that a holistic assessment of sleep patterns, rather than a single indicator, is key to predicting cognitive health in older adults. Unlike traditional linear assumptions, our study found a U-shaped association between sleep duration and cognitive function, with optimal nighttime sleep duration being 6–9 h, while napping for more than 90 min may have negative effects. In contrast to the public health recommendation that napping is universally beneficial, this study highlighted the ‘double-edged sword’ effect of napping, with a significant cognitive protection effect at nighttime sleeps for 6–9 h and naps for 61–90 min (OR = 0.65). This calls for a re-examination of the universality of sleep guidelines for the older adults.

This study provides a theoretical basis and practical tools for the early prevention and control of cognitive weakness in the older adults by revealing the complex interaction between sleep quality, duration and napping. We call for multidimensional sleep assessment to be included in the core content of geriatric health management, and to develop a hierarchical intervention strategy for high-risk groups to delay cognitive decline and improve quality of life.

There are also some limitations to this study. First of all, the evaluation scale used in the CHARLS database is a subjective evaluation scale, and no objective evaluation measures are used for its indicators. Secondly, the CHARLS sleep duration and sleep quality evaluation method was self-report, and there was a large degree of subjectivity in the self-evaluation of sleep duration and sleep quality. In the future, a cohort study is planned to carry out a prospective study to further analyze the combined effects of different levels, intensities and sleep duration on cognitive impairment in the older adults through objectively measured physical activity data, in order to provide more evidence-based basis for lifestyle intervention for cognitive impairment in the older adults.

## 5 Conclusion

Chinese older adults with poor sleep quality are at a 67% higher risk of cognitive weakness, and individuals with less than 6 h of sleep are at a 48% higher risk of cognitive weakness; No napping and excessive napping, the risk of cognitive debilitation increased by 23 and 69%, respectively. There is an additive interaction between sleep duration and quality and daytime napping on cognitive frailty in the older adults.

## Data Availability

The original contributions presented in the study are included in the article/Supplementary material, further inquiries can be directed to the corresponding author.
