# Supplementary material for: Association of sleep duration and sleep quality with cognitive frailty in Chinese older adults
Source: Front Public Health. 2025 Jun 6;13:1596965. doi: 10.3389/fpubh.2025.1596965 (PMC12178879; doi:10.3389/fpubh.2025.1596965)
Supplement: Supplementary file 1 [file Table_1.pdf]

Supplementary Table 1 sleep questionnaire

Variables	Problem	Data Type/Options	Example values
Nap	Whether the respondent had a napping habit	Categorical variables: 1=Yes，0=No	1（with nap）<br>0（no nap）
Nap-duration	If there is a nap, the average nap duration (in minutes)	Continuous value (minutes)	⩽60 min<br>61-90 min<br>> 90 min
Night sleep duration	Average nightly sleep duration per day (in hours)	Continuous values (hours)	⩽6 h<br>6-9 h<br>> 9 h
Sleep quality	Self-assessment of sleep quality	Categorical variables: 1=good,2=moderate, 3=poor	1（good）<br>2（moderate）<br>3（poor）
